# Supplementary material for: Machine learning and data-driven methods in computational surface and interface science
Source: NPJ Comput Mater. 2025 Jul 1;11(1):196. doi: 10.1038/s41524-025-01691-6 (PMC12213647; doi:10.1038/s41524-025-01691-6)
Supplement: Supplementary file 1 — Supplementary information [file 41524_2025_1691_MOESM1_ESM.pdf]

Machine Learning and Data-Driven Methods in Computational Surface and Interface Science

Lukas Hörmann[1,2*], Wojciech G. Stark[1] and Reinhard J. Maurer[1,2*]

[1*]Department of Chemistry, University of Warwick, Gibbet Hill Road, Coventry, CV4 7AL, United Kingdom.
[2*]Department of Physics, University of Warwick, Gibbet Hill Road, Coventry, CV4 7AL, United Kingdom.

*Corresponding author(s). E-mail(s): lukas.hoermann@warwick.ac.uk; r.maurer@warwick.ac.uk;

	**MD17**	**QM9**	**OC22**	**OC20 IS2RE**
DimeNet [1]	5.0, [2] 15.6 [3]	6.0, [4] 6.3 [5]	570.0, [6] 2475.1 [7]	661.3, [8] 683.5 [9]
PaiNN [10]	14.3, [3] 4.6* [11]	5.8 [4]	2630.0 [7]	743.0 [8]
SchNet [12]	6.9 [13]	14.0 [5]	7924.8 [7]	703.9, [4] 705.0 [8]
EquiformerV2 [14]		6.17 [14]	659.8 [14]	316.0 [14]
SpinConv [15]		12.0 [15]	1944.0 [7]	437.0, [14] 673.8 [8]
GemNet-dT [16]			1271.3 [7]	400.0 [14]
GemNet-OC [16]			828.7 [7]	344.0, [8] 355.0 [14]
NequIP [2]	4.2, [2] 0.7* [11]			736.0 [8]
Equiformer [4]		6.59 [4]		603.0, [17] 630.1 [4]
Faenet [18]		6.79 [18]		551.0 [19]
SEGNN [20]		15.0 [4]		679.0 [8]
SphereNet [21]		6.0 [4]		637.8 [8]
CGCNN [22]				851.0 [8]
Allegro [23]	0.8* [11]	4.7 [24]		
EAA [13]	5.9 [13]	12.0 [13]		
GM-sNN [25]	7.1, [13] 21.2 [3]	11.7 [25]		
MACE [11]	0.9* [11]	4.1 [24]		
PhysNet [26]	5.3 [27]	8.2 [5]		
TorchMD-NET [5]	3.6 [5]	6.2 [5]		
ANI [28]	21.7, [3] 8.8* [11]			
ACE [3]	2.0, [3] 2.2* [11]			
BOTNet [29]	0.7* [11]			
EANN [30]	6.4, [27] 6.5 [13]			
FCHL [31]	4.6, [27] 5.2, [3] 2.0* [11]			
GAP [32]	16.1, [3] 6.0* [11]			
NewtonNet [33]	5.0 [2]			
REANN [27]	4.4 [27]			
sGDML [34]	5.0, [27] 6.9 [3]			
So3krates [35]	4.3 [35]			
Cormorant [36]		22.0 [5]		
EGNN [37]		11.0 [5]		
EQGAT [38]		25.0 [4]		
LieConv [39]		19.0 [15]		
NMP [40]		20.0 [15]		

Supplementary Table 1 Mean absolute error (MAE/meV) for the prediction of energies (and geometry optimisations in case of OC20 IS2RE) using popular ML methods. ML methods trained and tested on the MD17/rMD17* database (1,000 training data points; rMD17 has improved convergence setting for the underlying training data), the QM9 database (110,000 training data points), the OC20 database (460,328 training data points), and the OC22 database (8,225,293 training data points).

References

[1] Gasteiger, J., Giri, S., Margraf, J.T., Günnemann, S.: Fast and Uncertainty-Aware Directional Message Passing for Non-Equilibrium Molecules (2022). https://arxiv.org/abs/2011.14115

[2] Batzner, S., Musaelian, A., Sun, L., Geiger, M., Mailoa, J.P., Kornbluth, M., Molinari, N., Smidt, T.E., Kozinsky, B.: E(3)-equivariant graph neural networks for data-efficient and accurate interatomic potentials. Nat. Commun. **13**(1), 1–11 (2022) https://doi.org/10.1038/s41467-022-29939-5

[3] Kovács, D.P., Oord, C.v.d., Kucera, J., Allen, A.E., Cole, D.J., Ortner, C., Csányi, G.: Linear atomic cluster expansion force fields for organic molecules: Beyond RMSE. J. Chem. Theory Comput. **17**(12), 7696–7711 (2021) https://doi.org/10.1021/acs.jctc.1c00647

[4] Liao, Y.-L., Smidt, T.: Equiformer: Equivariant Graph Attention Transformer for 3D Atomistic Graphs (2023). https://arxiv.org/abs/2206.11990

[5] Thölke, P., Fabritiis, G.D.: TorchMD-NET: Equivariant Transformers for Neural Network based Molecular Potentials (2022). https://arxiv.org/abs/2202.02541

[6] Lo, K., Huang, D.: On Training Derivative-Constrained Neural Networks (2023). https://arxiv.org/abs/2310.01649

[7] Tran, R., Lan, J., Shuaibi, M., Wood, B.M., Goyal, S., Das, A., Heras-Domingo, J., Kolluru, A., Rizvi, A., Shoghi, N., *et al.*: The open catalyst 2022 (oc22) dataset and challenges for oxide electrocatalysts. ACS Catal. **13**(5), 3066–3084 (2023) https://doi.org/10.1021/acscatal.2c05426

[8] Shoghi, N., Shoghi, P., Sriram, A., Das, A.: Distribution Learning for Molecular Regression (2024). https://arxiv.org/abs/2407.20475

[9] Chanussot, L., Das, A., Goyal, S., Lavril, T., Shuaibi, M., Riviere, M., Tran, K., Heras-Domingo, J., Ho, C., Hu, W., *et al.*: Open catalyst 2020 (oc20) dataset and community challenges. ACS Catal. **11**(10), 6059–6072 (2021) https://doi.org/10.1021/acscatal.0c04525

[10] Schütt, K., Unke, O., Gastegger, M.: Equivariant message passing for the prediction of tensorial properties and molecular spectra. In: Proceedings of the 38th International Conference on Machine Learning, pp. 9377–9388. PMLR, Honolulu, USA (2021). https://proceedings.mlr.press/v139/schutt21a.html Accessed 2022-02-09

[11] Batatia, I., Kovacs, D.P., Simm, G., Ortner, C., Csányi, G.: MACE: Higher order equivariant message passing neural networks for fast and accurate force fields. In: Koyejo, S., Mohamed, S., Agarwal, A., Belgrave, D., Cho, K., Oh, A. (eds.) Advances in Neural Information Processing Systems, vol. 35, pp. 11423–11436. Curran Associates, Inc., Red Hook, USA (2022). https://doi.org/10.48550/arXiv.2206.07697 . https://proceedings.neurips.cc/paper_files/paper/2022/file/4a36c3c51af11ed9f34615b81edb5bbc-Paper-Conference.pdf

[12] Schütt, K., Kindermans, P.-J., Sauceda Felix, H.E., Chmiela, S., Tkatchenko, A., Müller, K.-R.: Schnet: A continuous-filter convolutional neural network for modeling quantum interactions. In: Guyon, I., Luxburg, U.V., Bengio, S., Wallach, H., Fergus, R., Vishwanathan, S., Garnett, R. (eds.) Advances in Neural Information Processing Systems, vol. 30. Curran Associates, Inc., Red Hook, USA (2017)

[13] Chang, J., Kuai, Y., Wei, X., Yu, H., Lan, H.: Molecular potential energy computation via graph edge aggregate attention neural network. Chin. J. Chem. Phys. **36**(6), 691–699 (2023) https://doi.org/10.1063/1674-0068/cjcp2209136

[14] Liao, Y.-L., Wood, B., Das, A., Smidt, T.:

EquiformerV2: Improved Equivariant Transformer for Scaling to Higher-Degree Representations (2024). http://arxiv.org/abs/2306.12059 Accessed 2024-03-06

[15] Shuaibi, M., Kolluru, A., Das, A., Grover, A., Sriram, A., Ulissi, Z., Zitnick, C.L.: Rotation Invariant Graph Neural Networks using Spin Convolutions (2021). https://arxiv.org/abs/2106.09575

[16] Gasteiger, J., Becker, F., Günnemann, S.: Gemnet: Universal directional graph neural networks for molecules. Adv. Neural Inf. Process. Syst. **34**, 6790–6802 (2021)

[17] Kim, H., Woo, J., Kim, S., Moon, S., Kim, J.H., Kim, W.Y.: GeoTMI: predicting quantum chemical property with easy-to-obtain geometry via positional denoising. Adv. Neural Inf. Process. Syst. **36** (2024)

[18] Duval, A.A., Schmidt, V., Hernández-García, A., Miret, S., Malliaros, F.D., Bengio, Y., Rolnick, D.: FAENet: Frame averaging equivariant GNN for materials modeling. In: Krause, A., Brunskill, E., Cho, K., Engelhardt, B., Sabato, S., Scarlett, J. (eds.) Proceedings of the 40th International Conference on Machine Learning. Proceedings of Machine Learning Research, vol. 202, pp. 9013–9033. PMLR, Honolulu, USA (2023). https://proceedings.mlr.press/v202/duval23a.html

[19] Ramlaoui, A., Saulus, T., Terver, B., Schmidt, V., Rolnick, D., Malliaros, F.D., Duval, A.: Improving Molecular Modeling with Geometric GNNs: An Empirical Study (2024). https://arxiv.org/abs/2407.08313

[20] Yu, H., Zhong, Y., Hong, L., Xu, C., Ren, W., Gong, X., Xiang, H.: Spin-dependent graph neural network potential for magnetic materials. Phys. Rev. B **109**(14) (2024) https://doi.org/10.1103/physrevb.109.144426

[21] Coors, B., Condurache, A.P., Geiger, A.: Spherenet: Learning spherical representations for detection and classification in omnidirectional images. In: Proceedings of the European Conference on Computer Vision (ECCV), pp. 518–533 (2018)

[22] Xie, T., Grossman, J.C.: Crystal graph convolutional neural networks for an accurate and interpretable prediction of material properties. Phys. Rev. Lett. **120**(14), 145301 (2018) https://doi.org/10.1103/PhysRevLett.120.145301

[23] Musaelian, A., Batzner, S., Johansson, A., Sun, L., Owen, C.J., Kornbluth, M., Kozinsky, B.: Learning local equivariant representations for large-scale atomistic dynamics. Nat. Commun. **14**(1), 579 (2023) https://doi.org/10.1038/s41467-023-36329-y

[24] Shoghi, N., Kolluru, A., Kitchin, J.R., Ulissi, Z.W., Zitnick, C.L., Wood, B.M.: From Molecules to Materials: Pre-training Large Generalizable Models for Atomic Property Prediction (2024). https://arxiv.org/abs/2310.16802

[25] Zaverkin, V., Kästner, J.: Gaussian moments as physically inspired molecular descriptors for accurate and scalable machine learning potentials. J. Chem. Theory Comput. **16**(8), 5410–5421 (2020) https://doi.org/10.1021/acs.jctc.0c00347

[26] Unke, O.T., Meuwly, M.: PhysNet: A neural network for predicting energies, forces, dipole moments, and partial charges. J. Chem. Theory Comput. **15**(6), 3678–3693 (2019) https://doi.org/10.1021/acs.jctc.9b00181

[27] Zhang, Y., Xia, J., Jiang, B.: REANN: A PyTorch-based end-to-end multi-functional deep neural network package for molecular, reactive, and periodic systems. J. Chem. Phys. **156**(11) (2022)

[28] Smith, J.S., Isayev, O., Roitberg, A.E.: Ani-1: an extensible neural network potential with dft accuracy at force field computational cost. Chem. Sci. **8**, 3192–3203 (2017) https://doi.org/10.1039/C6SC05720A

[29] Batatia, I., Batzner, S., Kovács, D.P., Musaelian, A., Simm, G.N.C., Drautz, R., Ortner, C., Kozinsky, B., Csányi, G.: The

Design Space of E(3)-Equivariant Atom-Centered Interatomic Potentials (2022). https://doi.org/10.48550/arXiv.2205.06643 . https://arxiv.org/abs/2205.06643

[30] Zhang, Y., Hu, C., Jiang, B.: Embedded atom neural network potentials: Efficient and accurate machine learning with a physically inspired representation. J. Phys. Chem. Lett. **10**(17), 4962–4967 (2019)

[31] Faber, F.A., Christensen, A.S., Huang, B., Von Lilienfeld, O.A.: Alchemical and structural distribution based representation for universal quantum machine learning. J. Chem. Phys. **148**(24), 241717 (2018) https://doi.org/10.1063/1.5020710

[32] Bartók, A.P., Payne, M.C., Kondor, R., Csányi, G.: Gaussian approximation potentials: The accuracy of quantum mechanics, without the electrons. Phys. Rev. Lett. **104**(13), 136403 (2010) https://doi.org/10.1103/PhysRevLett.104.136403

[33] Haghighatlari, M., Li, J., Guan, X., Zhang, O., Das, A., Stein, C.J., Heidar-Zadeh, F., Liu, M., Head-Gordon, M., Bertels, L., *et al.*: NewtonNet: a Newtonian message passing network for deep learning of interatomic potentials and forces. Digit. Discov. **1**(3), 333–343 (2022) https://doi.org/10.1039/D2DD00008C

[34] Chmiela, S., Sauceda, H.E., Müller, K.-R., Tkatchenko, A.: Towards exact molecular dynamics simulations with machine-learned force fields. Nat. Commun. **9**(1), 1–10 (2018) https://doi.org/10.1038/s41467-018-06169-2

[35] Frank, T., Unke, O., Müller, K.-R.: So3krates: Equivariant attention for interactions on arbitrary length-scales in molecular systems. In: Koyejo, S., Mohamed, S., Agarwal, A., Belgrave, D., Cho, K., Oh, A. (eds.) Advances in Neural Information Processing Systems, vol. 35, pp. 29400–29413. Curran Associates, Inc., Red Hook, USA (2022)

[36] Anderson, B., Hy, T.S., Kondor, R.: Cormorant: Covariant molecular neural networks. Adv. Neural Inf. Process. Syst. **32** (2019)

[37] Satorras, V.G., Hoogeboom, E., Welling, M.: E(n) equivariant graph neural networks. In: Meila, M., Zhang, T. (eds.) Proceedings of the 38th International Conference on Machine Learning. Proceedings of Machine Learning Research, vol. 139, pp. 9323–9332. PMLR, Honolulu, USA (2021). https://proceedings.mlr.press/v139/satorras21a.html

[38] Le, T., Noé, F., Clevert, D.-A.: Equivariant Graph Attention Networks for Molecular Property Prediction (2022). https://arxiv.org/abs/2202.09891

[39] Finzi, M., Stanton, S., Izmailov, P., Wilson, A.G.: Generalizing convolutional neural networks for equivariance to lie groups on arbitrary continuous data. In: III, H.D., Singh, A. (eds.) Proceedings of the 37th International Conference on Machine Learning. Proceedings of Machine Learning Research, vol. 119, pp. 3165–3176. PMLR, Honolulu, USA (2020). https://proceedings.mlr.press/v119/finzi20a.html

[40] Gilmer, J., Schoenholz, S.S., Riley, P.F., Vinyals, O., Dahl, G.E.: Neural message passing for quantum chemistry. In: Precup, D., Teh, Y.W. (eds.) Proceedings of the 34th International Conference on Machine Learning. Proceedings of Machine Learning Research, vol. 70, pp. 1263–1272. PMLR, Honolulu, USA (2017). https://proceedings.mlr.press/v70/gilmer17a.html
